# Prediction of microvascular obstruction by coronary artery angiography score after acute ST-segment elevation myocardial infarction: a single-center retrospective observational study

**DOI:** 10.1186/s12872-022-02836-x

**Published:** 2022-09-14

**Authors:** Ziwen Li, Hongbin Yin, Dehua Wang, Yayun Zhang, Yongli Feng, Yi Zhou, Ying Zhou

**Affiliations:** 1 Lianyungang Clinical Medical College of Nanjing Medical University, Lianyungang, Jiangsu China; 2 Lianyungang Clinical Medical College of Jinzhou Medical University, Lianyungang, Jiangsu China; 3 Department of Radiology, The First Affiliated Hospital of Kangda College Nanjing Medical University, Lianyungang, Jiangsu China; 4 Department of Cardiology, Lianyungang First People’s Hospital, Lianyungang, Jiangsu China

**Keywords:** Cardiac magnetic resonance, Acute ST-segment elevation myocardial infarction, Microvascular occlusion, SYNTAX score, Gensini score

## Abstract

**Background:**

Some coronary artery angiography (CAG) scores are associated with the no-reflow phenomenon after percutaneous coronary intervention (PCI) in patients with acute ST-segment elevation myocardial infarction (STEMI). However, quality evidence regarding the association between the CAG scores and microvascular injury is still needed. Our study aimed to validate the ability of the CAG scores in predicting microvascular obstruction (MVO) detected by cardiac magnetic resonance (CMR) imaging.

**Methods:**

From October 2020 to October 2021, 141 consecutive patients with acute STEMI who underwent primary PCI and CMR were retrospectively reviewed. CMR imaging was performed between 3 and 7 days after PCI. The patients were divided into MVO and non-MVO group based on the CMR results. Three CAG scores (SYNTAX score, SYNTAX II score and Gensini score) were used to assess the severity of coronary artery atherosclerotic burden.

**Results:**

A total of 122 patients were included (mean age 60.6 ± 12.8 years). MVO occurred in 51 patients (41.8%). Patients with MVO had higher SYNTAX scores, SYNTAX II scores and Gensini scores than those without MVO (all *p* < 0.001). The Gensini score (*r* = 0.567, *p* < 0.001) showed the strongest correlation with infarction size than SYNTAX score (*r* = 0.521, *p* < 0.001) and SYNTAX II score (*r* = 0.509, *p* < 0.001). The areas under the receiver operator characteristic curves of SYNTAX score, SYNTAX II score and Gensini score for predicting MVO patients were 0.726, 0.774 and 0.807. In multivariable regression analysis, peak troponin I (odd ratio [OR] = 1.236, *p* = 0.001) and SYNTAX II score (OR = 11.636, *p* = 0.010) were identified as independent predictors of MVO.

**Conclusions:**

In patients with acute STEMI undergoing primary PCI treatment, the peak troponin I and SYNTAX II score may be an independent predictor of MVO.

## Background

Early percutaneous coronary intervention (PCI) has been reported to significantly reduce mortality in patients with acute ST-segment elevation myocardial infarction (STEMI) [1]. However, the consequent improvement in myocardial perfusion post-PCI is not universal. About 50% of patients undergoing primary PCI shows delayed myocardial perfusion [2, 3]. This phenomenon is called as microvascular obstruction (MVO) and is related to structural and functional damage at the microvascular level caused by PCI revascularization [4, 5]. Previous studies have shown a significant increase in the incidence of major adverse cardiovascular events (MACE) in patients with MVO [3].

Cardiac magnetic resonance (CMR) is a non-invasive imaging tool with high spatial resolution, has been widely used to detect and quantify the extent of MVO [6–8]. However, the long breath-holding requirements make it difficult for STEMI patients to cooperate, and excessive dosage of gadolinium contrast agent increases the risk of renal failure.

Some coronary artery angiography (CAG) scores, such as the SYNTAX score [9] and Gensini score [10], have been used to assess the coronary artery atherosclerotic burden. SYNTAX score has an excellent performance in predicting the MACE in STEMI patients after PCI [11, 12]. More recently, it has been shown that the SYNTAX score and Gensini score are associated with the no-reflow phenomenon (thrombolysis in myocardial infarction [TIMI] blood flow < 3 or myocardial color grade < 2) after PCI [13, 14]. However, quality evidence regarding the association between the CAG scores and microvascular injury in acute STEMI patients is still needed. In addition, it has been shown that the SYNTAX II score’s sensitivity and specificity are higher than SYNTAX score in predicting in-hospital mortality of high-risk patients with STEMI [15]. The SYNTAX II score connects the characteristics of clinical variables with coronary anatomy, and may also have an important role in predicting microvascular injury. Therefore, our study aimed to validate the ability of three CAG scores in predicting MVO detected by CMR imaging.

## Methods

### Study population

This retrospective single-center cohort study enrolled 141 consecutive patients with acute STEMI who underwent primary PCI and CMR at Lianyungang First People’s Hospital between October 2020 and October 2021. STEMI was defined as a combination of chest pain suggested that the duration of myocardial ischemia was > 30 min. The electrocardiography (ECG) indicated ST segment elevation > 2 mm on at least two precordial leads or limb leads, and troponin levels significantly increased beyond double the normal upper limit. The inclusion criteria were as follows: (1) diagnosis of STEMI; (2) PCI performed within 12 h of symptom onset; (3) CAG performed before and immediately after PCI; and (4) CMR performed between 3 and 7 days after PCI. The exclusion criteria were as follows: (1) age < 18 years; (2) previous history of myocardial infarction or revascularization (n = 10); and (3) incomplete CAG image (n = 6) or poor CMR image quality (n = 3). Finally, 122 patients were enrolled (mean age 60.6 ± 12.8 years old; 72.1% was male).

### PCI and CAG

PCI treatment was performed using Seldinger's method in all patients with a median time of 4.5 h, using a 5F contrast catheter, 6F guide catheter, and 0.014-inch guide wire. A total of 100 ml nonionic contrast agent (Ultravist 370; Bayer AG, Berlin, Germany) was injected. Heparin was intravenously injected (7.5 ml) and maintained (17.5 ml/h). Drug-eluting stents were used in all the patients. All stents were implanted directly whenever possible, and balloon dilation angioplasty (8–10 atmospheric pressure) was performed in the remaining cases. TIMI blood flow grades were recorded before and after PCI.

### CAG score

Based on the results of coronary artery angiography before PCI, three CAG scores (SYNTAX score, SYNTAX II score and Gensini score) were used to assess the severity of coronary artery atherosclerotic burden by two uninformed cardiologists. The SYNTAX score and SYNTAX II score were calculated using the SYNTAX score online calculator (http://www.SYNTAXscore.com). The SYNTAX score evaluated all coronary lesions with diameter stenosis > 50% and vessels with diameter > 1.5 mm [16]. The SYNTAX score for the degree of coronary stenosis were 2 for 50–99%, 5 for 100%. The SYNTAX II score was composed of SYNTAX score and six clinical variables, including age, gender, creatinine clearance, LVEF, chronic obstructive pulmonary disease, and peripheral arterial disease. The Gensini score was calculated based on the product of the degree scoring of coronary stenosis and the location score of the lesion [10]. The Gensini score for the degree of coronary stenosis were 1 for 1–25%, 2 for 26–50%, 4 for 51–75%, 8 for 76–90%, 16 for 91–99%, 32 for 100%. The culprit artery was defined as the target artery treated with PCI.

### Cardiac magnetic resonance

CMR examinations were performed in all patients after PCI with a median time of 5.2 days. CMR was performed with a 1.5 T MR scanner (Magnetom Aera XJ, Siemens, Erlangen, Germany) using an 12-element phased-array coil equipped with ECG-gated. The cardiac cine imaging was performed using the steady-state free precession sequence (SSFP) sequence; slice gap, 0 mm; slice thickness, 8 mm; repetition time, 37 ms; echo time, 1.2 ms; field of view, 340 × 340 mm^2^. T2-weighted imaging was performed using the short time inversion recovery (STIR) sequence with the same prescription of cine images. Late gadolinium enhancement (LGE) imaging was performed using the phase sensitive inversion recovery (PSIR) sequence; slice gap, 0 mm; slice thickness, 8 mm; echo time, 1.3 ms; inversion time, 300–340 ms; field of view, 340 × 340 mm^2^.

All images were evaluated on the CMR post-processing software (cvi42 v5.13, Circle Cardiovascular Imaging, Calgary, Canada). CMR images were interpreted by two uninformed radiologists, and consensus was reached when opinions were different. Left ventricular ejection fraction (LVEF) were calculated from cine images. The area at risk (AAR) was defined as the high-signal region (> 2 standard deviations [SD] of the normal myocardial signal) on T2-weighted images. T2-weighted images with poor image quality or unrecognizable AAR area were removed. The infarction size (IS) was quantified on LGE images 10 min after gadolinium injection (Gadoteric Acid Meglumine Salt Injection, Hengrui, Lianyungang, China). The signal threshold for IS was set to > 5 SD of the normal myocardial signal. Myocardial salvage index (MSI) was calculated as: MSI = (AAR − IS)/AAR × 100. The MVO was defined as a hypoenhanced region within LGE area. AAR and IS were recorded as percentage of the left ventricular (LV).

### Statistical analysis

Statistical analyses were performed using the IBM SPSS software (version 26.0; IBM Corp, Chicago, IL, USA). Continuous variables were expressed as mean ± SD or median (25th to 75th percentile), and were compared using independent sample t-tests or Mann–Whitney U-tests. Categorical data were expressed as n (%) and were compared using Chi-squared tests. Spearman correlation analysis was used to assess the correlation between coronary scores and IS (%LV). The area under the curve (AUC) in the receiver operating characteristic (ROC) analysis was used to assess predictive performance. Binary logistic regression analysis was performed to assess the coronary scores for predicting MVO (results were expressed as odds ratio [OR] and 95% confidence interval [CI]). Coronary scores were converted into categorical variables based on the cutoff value. The variables of *p* < 0.1 in the univariable analysis were included in the multivariable tests. All tests were two-tailed and *p* < 0.05 was considered as statistically significant.

## Results

### Characteristics of patients

**Fig. 1:**
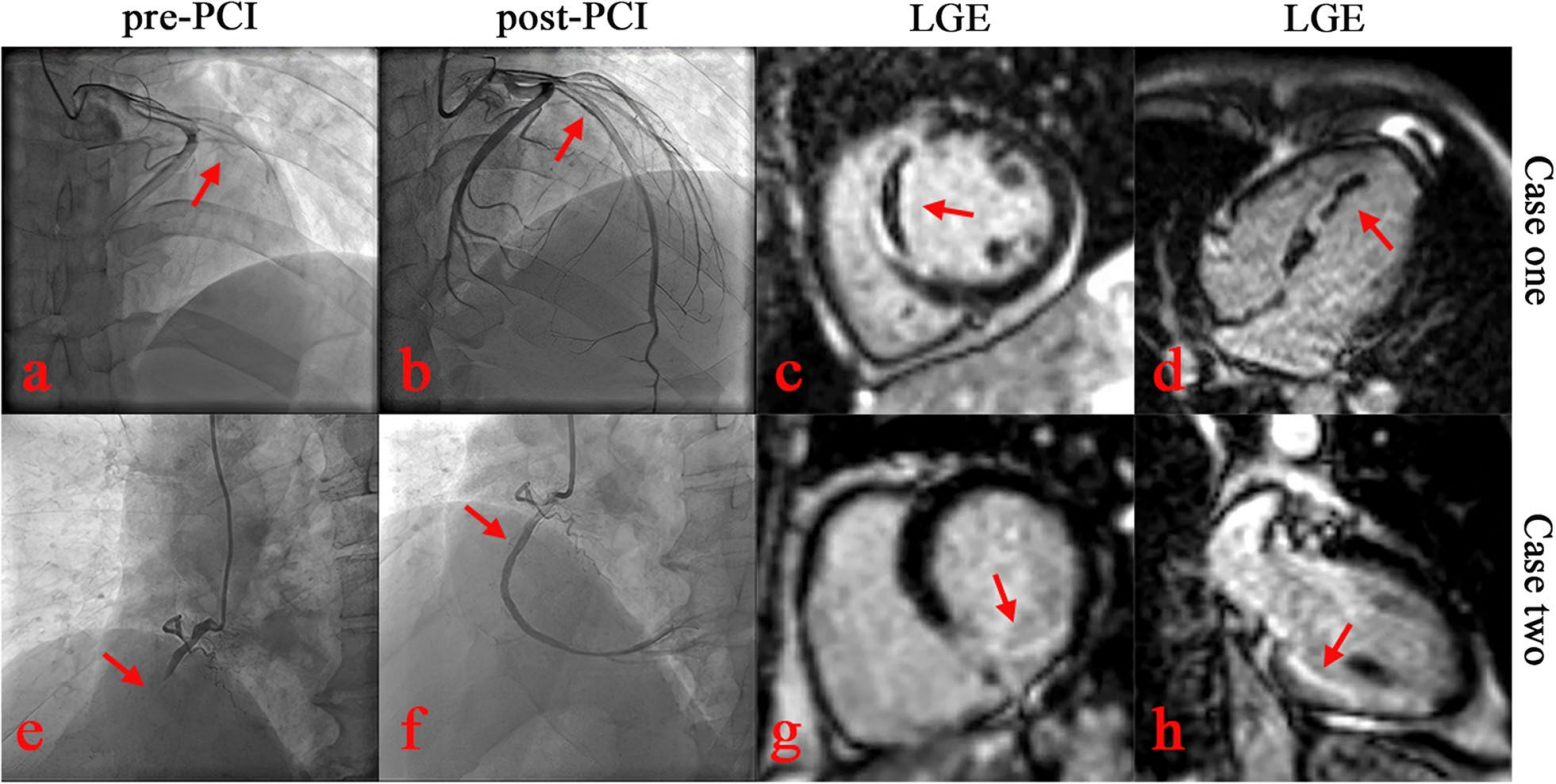
Two typical examples (with and without MVO) of angiography and cardiac magnetic resonance image. Case one (**a–d**): A 49-year-old male STEMI patient with MVO was diagnosed to have a total occluded proximal segment of the LAD (SYNTAX score 20.5, SYNTAX II score 39, and Gensini score 80); Case two (**e–h**): A 68-year-old male STEMI patient without MVO was diagnosed to have a total occluded proximal segment of the RCA (SYNTAX score 10, SYNTAX II score 30.6, and Gensini score 32). *STEMI* ST-segment elevation myocardial infarction; *MVO* microvascular obstruction; *LAD* left anterior descending artery; *RCA* right coronary artery

A total of 122 patients with acute STEMI were enrolled, with 51 (41.8%) in the MVO group and 71 (58.2%) in the non-MVO group. Typical examples (with and without MVO) of patients are shown in Fig. 1. In the comparison of data between the two groups, there were no statistically significant differences in age, gender, heart rate, systolic blood pressure, diastolic blood pressure, hypertension, diabetes, current Smoker, dyslipidemia, GRACE score, serum creatinine, serum uric acid, albumin, plasma fibrinogen, D-Dimer, neutrophil, lymphocyte, total cholesterol, triglyceride, low density lipoprotein cholesterol (LDL-C), high density lipoprotein cholesterol (HDL-C), Killip class, time from symptom onset to PCI, time from PCI to CMR, culprit artery left circumflex artery (LCX), number of diseased arteries, and final TIMI flow grade (all *p* > 0.05). Compared with the non-MVO group, the MVO group had higher peak Troponin I, higher peak creatine kinase (CK), higher peak creatine kinase isoenzyme (CK-MB), higher pro-B-type natriuretic peptide (PRO-BNP), higher C-reaction protein (CPR), more culprit artery left anterior descending artery (LAD), less culprit artery right coronary artery (RCA), more initial TIMI 0/1 flow, lower LVEF, larger AAR (%LV), larger and IS (%LV), lower MSI, higher SYNTAX scores, higher SYNTAX II scores and higher Gensini scores (all *p* < 0.05) (Table 1).

**Table 1:** Characteristics of patients

Parameters	Total population (n = 122)	Non-MVO group (n = 71)	MVO group (n = 51)	*p*-value
Age (years)	60.6 ± 12.8	61.1 ± 14.1	59.8 ± 10.9	0.593
Male, *n* (%)	88 (72.1)	49 (69.0)	39 (76.5)	0.365
Heart rate (bpm)	79.8 ± 13.7	76.8 ± 12.9	82.9 ± 18.5	0.154
Systolic blood pressure (mmHg)	133.5 ± 20.7	133.0 ± 23.9	134.2 ± 15.5	0.733
Diastolic blood pressure (mmHg)	81.6 ± 14.1	80.6 ± 14.7	83.0 ± 13.2	0.345
Hypertension, *n* (%)	58 (47.5)	36 (50.7)	22 (43.1)	0.409
Diabetes, *n* (%)	32 (26.2)	17 (23.9)	15 (29.4)	0.498
Current Smoker, *n* (%)	27 (22.1)	13 (18.3)	14 (27.5)	0.230
Dyslipidemia, *n* (%)	16 (13.1)	10 (14.1)	6 (11.8)	0.708
GRACE score, *n* (%)	141.8 ± 29.0	133.2 ± 23.9	140.1 ± 31.2	0.188
Peak Troponin I (ng/ml)	23.7 (13.7 to 30.0)	15.8 (9.8 to 28.6)	30.0 (23.7 to 30.0)	< 0.001
Peak CK (U/L)	2748.5 (1432.5 to 3817.5)	1925.5 (1138.5 to 3053.0)	3505.0 (2708.5 to 6920.3)	< 0.001
Peak CK-MB (U/L)	270.0 (202.0 to 404.0)	211.0 (163.0 to 276.0)	346.0 (274.0 to 473.0)	< 0.001
PRO-BNP (pg/mL)	815.0 (432.5 to 1996.5)	683.5 (427.8 to 933.0)	987.0 (515.8 to 3205.3)	0.026
CRP (mg/L)	6.9 (3.2 to 17.1)	4.8 (3.0 to 13.5)	9.9 (4.2 to 25.9)	0.008
Serum creatinine (umol/L)	58.6 (49.9 to 75.6)	58.4 (48.7 to 72.0)	58.9 (52.0 to 79.8)	0.144
Serum uric acid (umol/L)	301.2 (242.2 to 383.1)	291.6 (236.1 to 362.6)	307.8 (244.9 to 405.5)	0.234
Albumin (g/L)	37.3 ± 4.3	37.1 ± 4.4	37.6 ± 4.0	0.592
Plasma fibrinogen (g/L)	2.6 ± 1.0	2.6 ± 1.0	2.6 ± 0.9	0.882
D-Dimer (ng/ml)	89.7 (67.4 to 154.5)	84.7 (67.5 to 125.0)	99.0 (67.1 to 187.0)	0.075
Neutrophil (× 10^9^/L)	7.9 (6.3 to 10.3)	7.9 (6.1 to 9.8)	8.5 (6.3 to 10.7)	0.165
Lymphocyte (× 10^9^/L)	1.3 (0.9 to 1.6)	1.2 (0.9 to 1.6)	1.3 (1.0 to 1.5)	0.682
Total cholesterol (mmol/L)	4.1 (3.6 to 4.8)	4.2 (3.6 to 4.8)	3.9 (3.5 to 4.9)	0.267
Triglyceride (mmol/L)	1.2 (0.8 to 1.6)	1.1 (0.8 to 1.6)	1.4 (0.9 to 1.7)	0.075
LDL-C (mmol/L)	2.5 ± 0.6	2.5 ± 0.7	2.5 ± 0.5	0.458
HDL-C (mmol/L)	1.2 (1.0 to 1.6)	1.2 (1.0 to 1.6)	1.2 (1.0 to 1.5)	0.769
*Heart failure Killip class, n (%)*				
I	91 (74.6)	55 (77.5)	36 (70.6)	0.389
≥ II	31 (15.4)	16 (12.5)	15 (29.4)	
Time from symptom onset to PCI (h)	6.2 (4.5 to 9.3)	6.0 (5.0 to 9.0)	6.2 (4.0 to 11.3)	0.545
*Culprit artery, n (%)*				
LAD	66 (54.1)	28 (39.4)	38 (74.5)	< 0.001
LCX	9 (7.4)	6 (8.5)	3 (5.9)	0.592
RCA	47 (38.5)	37 (52.1)	10 (19.6)	< 0.001
*Number of diseased arteries, n (%)*				
Single-artery	64 (52.5)	34 (47.9)	30 (58.8)	0.233
Multi-artery	58 (47.5)	37 (42.1)	21 (41.2)	
*Initial TIMI flow grade, n (%)*				
0/1	95 (77.9)	48 (67.6)	47 (92.2)	0.001
2/3	27 (67.6)	23 (32.4)	4 (7.8)	
*Final TIMI flow grade, n (%)*				
0/1	112 (91.8)	67 (94.4)	45 (88.2)	0.223
2/3	10 (8.2)	4 (5.6)	6 (11.8)	
Time from PCI to CMR (days)	5.2 (4.3 to 6.4)	5.1 (3.8 to 6.2)	5.2 (4.5 to 6.9)	0.275
LVEF (%)	36.7 ± 10.2	40.7 ± 9.7	31.2 ± 8.4	< 0.001
AAR (% LV)	28.3 (22.5 to 34.8)	25.3 (20.4 to 29.3)	34.8 (28.2 to 37.8)	< 0.001
IS (% LV)	22.3 (15.6 to 32.5)	18.4 (13.2 to 22.3)	27.3 (23.8 to 33.1)	< 0.001
MSI (%)	22.3 (13.9 to 37.2)	25.7 (14.9 to 44.8)	16.3 (8.8 to 22.2)	< 0.001
SYNTAX score	17.8 (11.0 to 22.0)	16.0 (10.0 to 18.0)	20.0 (15.8 to 22.3)	< 0.001
SYNTAX II score	33.6 ± 10.0	29.8 ± 8.8	38.8 ± 9.2	< 0.001
Gensini score	53.0 (36.0 to 80.0)	44.0 (32.0 to 58.0)	74.0 (50.5 to 81.5)	< 0.001

Values are mean ± SD, number (%) or median (25th to 75th percentile)

*MVO* Microvascular obstruction; *GRACE* The global registry of acute coronary events; *CK* Creatine kinase; *CK-MB* Creatine kinase isoenzyme; *PRO-BNP* Pro-B-type natriuretic peptide; *CRP* C-reaction protein; *LDL-C* Low density lipoprotein cholesterol; *HDL-C* High density lipoprotein cholesterol; *PCI* Percutaneous coronary intervention; *LAD* Left anterior descending coronary artery; *LCX* Left circumflex coronary artery; *RCA* Right coronary artery; *TIMI* Thrombolysis in myocardial infarction; *CMR* Cardiac magnetic resonance; *LVEF* Left ventricular ejection fraction; *AAR* Area at risk; *IS* Infarct size; *MSI* Myocardial salvage index

### Correlations between coronary scores and IS

**Fig. 2:**
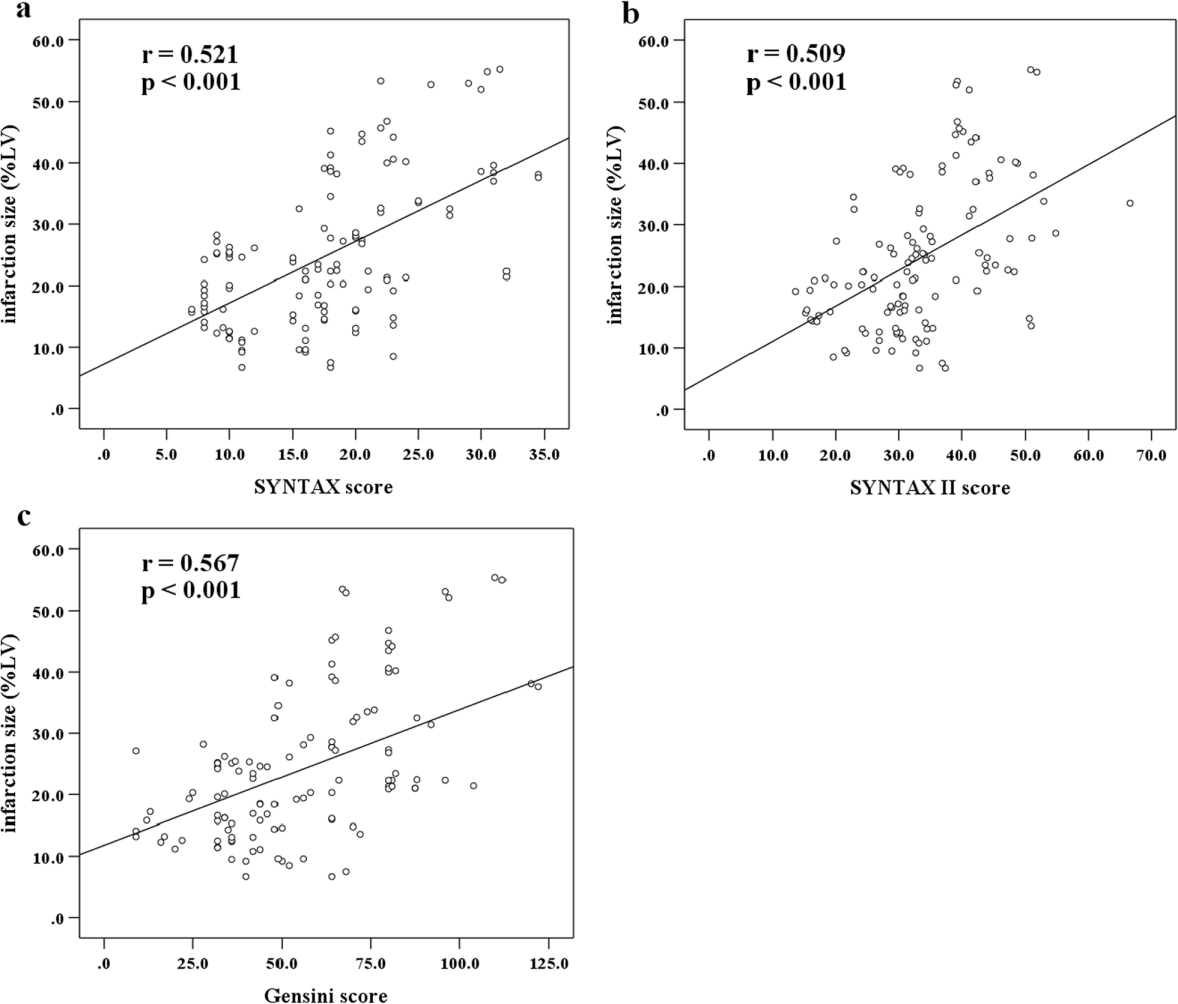
Correlations of the SYNTAX score **a**, SYNTAX II score **b** and Gensini score **c** with infarct size (%LV). *LV* left ventricular

In the correlation analysis, three coronary scores were positively correlated with IS (%LV). Among them, Gensini score (*r* = 0.567, *p* < 0.001) showed the strongest correlation with IS (%LV) than SYNTAX score (*r* = 0.521, *p* < 0.001) and SYNTAX II score (*r* = 0.509, *p* < 0.001) (Fig. 2).

### ROC curve analysis

**Fig. 3:**
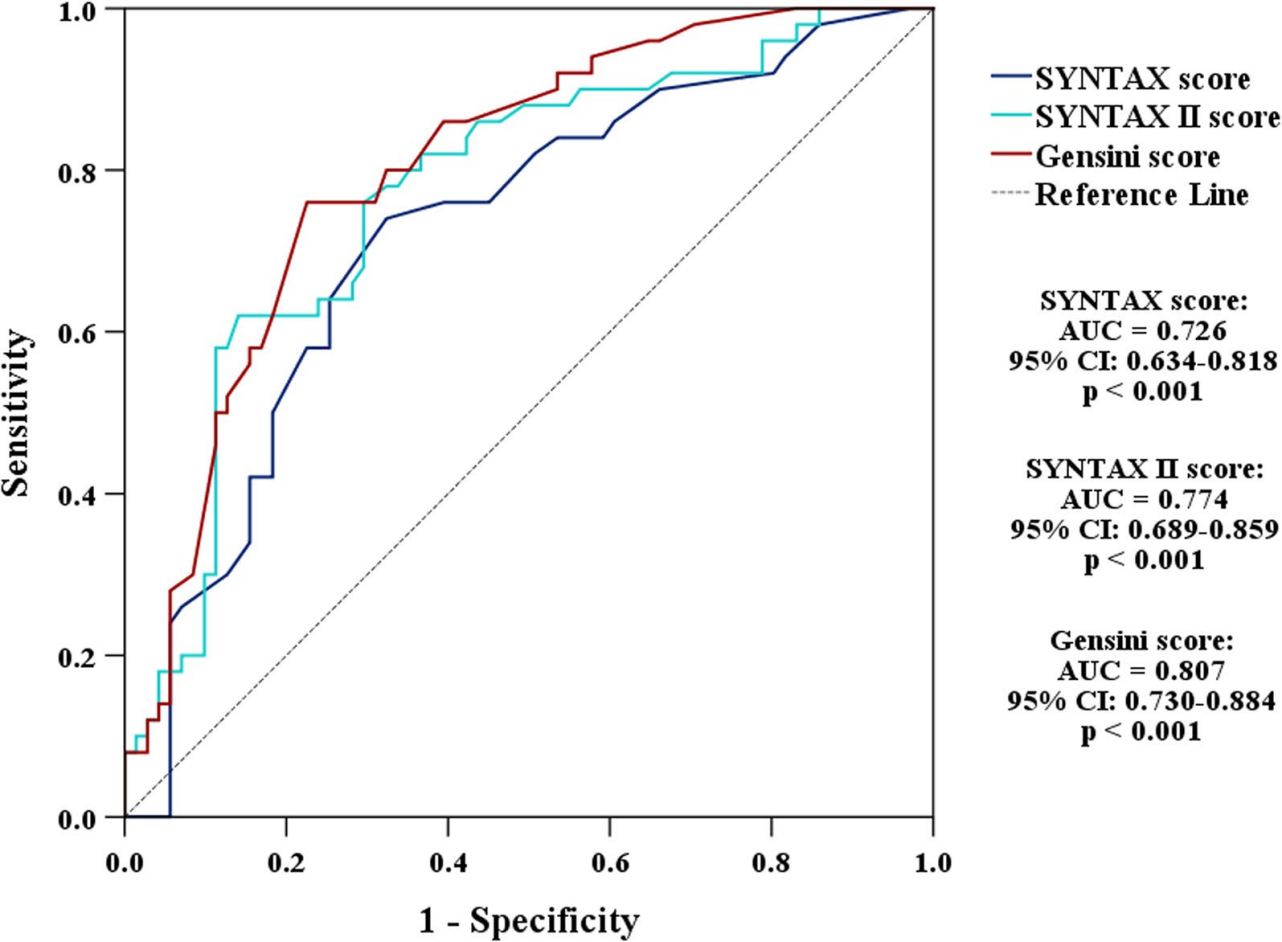
Receiver operating characteristic (ROC) curves for coronary scores to predict microvascular obstruction

The ROC curve analysis of the three coronary scores predicted MVO is shown in Table 2. Among them, Gensini score showed the largest area under the curve (AUC = 0.807, 95% CI 0.730–0.884, *p* < 0.001) than SYNTAX score (AUC = 0.726, 95% CI 0.634–0.818, *p* < 0.001) and SYNTAX II score (AUC = 0.774, 95% CI 0.689–0.859, *p* < 0.001). At the optimal cutoff value of 61, the Gensini score predicted MVO sensitivity and specificity were 76.5% and 77.5%. At the optimal cutoff value of 17.5, the SYNTAX score predicted MVO sensitivity and specificity were 74.5% and 67.6%. At the optimal cutoff value of 36.4, the SYNTAX II score predicted MVO sensitivity and specificity were 62.7% and 85.9% (Fig. 3).

**Table 2:** Univariable and multivariable regression analysis for MVO

Variable	OR	95% CI	*p*-value
*Univariable analysis*			
Peak Troponin I	1.153	1.089–1.220	< 0.001
Peak CK	1.000	1.000–1.001	< 0.001
Peak CK-MB	1.003	1.001–1.005	0.005
PRO-BNP	1.001	1.001–1.006	0.006
CRP	1.012	0.997–1.027	0.132
Culprit artery LAD	4.489	2.039–9.885	< 0.001
Culprit artery RCA	0.224	0.097–0.516	< 0.001
Initial TIMI flow 0/1	5.630	1.809–17.524	0.003
LVEF (%)	0.895	0.854–0.939	< 0.001
AAR (% LV)	1.107	1.043–1.175	0.001
IS (% LV)	1.221	1.138–1.311	< 0.001
MSI (%)	0.927	0.885–0.971	0.001
SYNTAX score	6.100	2.735–13.607	< 0.001
SYNTAX II score	10.274	4.274–24.698	< 0.001
Gensini score	11.172	4.758–26.231	< 0.001
*Multivariable analysis*			
Peak Troponin I	1.236	1.095–1.395	0.001
SYNTAX II score	11.636	1.816–74.556	0.010

*MVO* Microcirculation obstruction; *OR* Odd ratio; *CI* Confidence interval; *CK* Creatine kinase; *CK-MB* Creatine kinase isoenzyme; *PRO-BNP* Pro-B-type natriuretic peptide; *CRP* C-Reaction protein; *LAD* Left anterior descending coronary artery; *RCA* Right coronary artery; *TIMI* Thrombolysis in myocardial infarction; *LVEF* Left ventricular ejection fraction; *AAR* Area at risk; *IS* Infarction size

### Univariable and multivariable analysis

In the univariable analysis for predicting MVO, variables including peak troponin I, peak CK, Peak CK-MB, culprit artery LAD, culprit artery RCA, Initial TIMI flow 0/1, LVEF, AAR, IS, MSI, SYNTAX score, SYNTAX II score, and Gensini score had a *p*-value < 0.1 After adjustment for other potential confounders including PRO-BNP, AAR, IS and MSI, the multivariable analysis showed that the peak troponin I (OR = 1.236, 95% CI 1.095—1.395, *p* = 0.001) and SYNTAX II score (OR = 11.636, 95% CI 1.816—74.556, *p* = 0.010) were identified as independent predictors of MVO (Table 2).

## Discussion

In the present study, we investigated the association between the CAG scores and MVO in patients with acute STEMI after PCI. We found that (1) patients with MVO had higher SYNTAX score, SYNTAX II score, and Gensini score than those without MVO; (2) Gensini score showed the strongest correlation with IS than SYNTAX score and SYNTAX II score; and (3) the peak troponin I and SYNTAX II score were independent associated with MVO after adjustment for potential confounders.

Coronary artery angiography score is widely used to assess extent and severity of coronary atherosclerotic disease (CAD) burden [17]. Previous studies proved that the SYNTAX score and Gensini score could predict the no-reflow phenomenon defined by TIMI flow or myocardial color grade post-PCI [13, 14]. However, multiple evidences showed that TIMI flow or myocardial color grade underestimated the prevalence of microvascular injury [2, 18]. CMR is an excellent imaging tool for detecting MVO with high sensitivity and specificity, and a recent expert consensus recommends 3–7 days post-PCI as the preferred CMR imaging time point to evaluate MVO [19]. Therefore, evaluating the association between coronary scores and MVO can provide higher quality evidence for early identification of patients with microvascular injury.

The MVO is a severe marker of microvascular blood flow dysfunction. The development of MVO depends on several factors: (1) the shedding of atherosclerotic or thrombotic debris caused by PCI [20], (2) vasospasm caused by the release of vasoconstrictor factors such as endothelin and thromboxane [21], (3) activation of platelets and coagulation factors in microvascular caused by endothelial cell injury [4], and (4) progressive inflammatory cells accumulation in the infarct area [22]. Our study showed that patients with MVO had significantly higher coronary scores than those without MVO. This may be because higher coronary scores indicated that the coronary artery stenosis was more severe and the lesion site was closer to the proximal segment of the LV dominant artery, which also suggested a larger area of LV myocardium with insufficient blood supply [23]. Previous studies also proved that the development of MVO was associated with reduced initial TIMI grade, anterior infarct location, and larger IS [2, 3]. Therefore, these reason may explain the association between MVO and higher coronary scores.

The SYNTAX score [11, 12] and SYNTAX II score [15, 24] have excellent performance in predicting the mortality and MACE during hospitalization and follow-up in high-risk patients after PCI. However, our results showed that the Gensini score had the largest AUC in predicting MVO than the SYNTAX score and SYNTAX II score. Although three scores were grading the severity of CAD, there are many differences between them. In the SYNTAX score algorithm, the percent diameter stenosis was not carefully differentiated, and only non-occlusive (50–99% diameter stenosis) and occlusive (100% diameter stenosis) lesion were considered. In addition, excessive morphological scores may weakened the effect of thrombotic load in patients with acute STEMI. In the Gensini score algorithm, the percent diameter stenosis was carefully differentiated from 25 to 100%, and the severity score of lesion stenosis increased geometrically, which reinforced the effect of thrombotic load in patients with acute STEMI.

In the univariable analysis, three coronary scores were predictors of MVO. Interestingly, when the SYNTAX II score was added to the multivariable analysis, the Gensini score and SYNTAX score lost their predictive value. This is most likely related to the role of some clinical variables in the present study. SYNTAX II Score is a comprehensive score that connects coronary anatomy with clinical variables such as age, gender, creatinine clearance, LVEF, etc. Previous studies proved that apart from haemodynamic deterioration, some clinical parameters such as chronic renal failure, lactate, and LVEF had an impact on in-hospital mortality in high-risk patients after PCI [25, 26]. Moreover, the occurrence of MVO is closely related to the decreased LVEF and the increased inflammatory markers [2, 27], as our results demonstrated. Therefore, the SYNTAX II score including LVEF and others clinical variables performed better in predicting MVO.

The CAG scores has also been associated with the magnitude of myocardial injury. Several studies have shown that the SYNTAX score correlated signifcantly with biomarkers of cardiac injury [28, 29]. In a CMR study, Gao et al. [23] observed SYNTAX score was signifcant positive correlation with IS and was the independent predictor of high IS (≥ mean 35.43%LV). These findings were consistent with those of our study. Furthermore, in our study, Gensini score showed the strongest correlation with IS (%LV) than other scores. Previous studies showed that Gensini score was correlated with atherosclerotic plaque burden assessed by intracoronary ultrasound [30]. Therefore, the Gensini score could be a more simple and effective tool for infarct severity assessment post-STEMI.

The study had several limiations. First, this was a single-center study with a small sample size. A multi-center studies with larger sample size would be required reconfirm our results. Second, The observational retrospective design of this study may have resulted in bias in information selection. Finally, this study used angiography-based visual scoring to predict MVO and the results may be affected by observer subjective factors.

## Conclusion

In patients with acute STEMI undergoing primary PCI treatment, the peak troponin I and SYNTAX II score may be an independent predictor of MVO.

## Data Availability

The data set supporting the results of this article are included within the article. The datasets used and/or analyzed during the current study are available from the corresponding author on reasonable request.

## Abbreviations

STEMI: ST-segment elevation myocardial infarction
PCI: Percutaneous coronary intervention
MVO: Microvascular obstruction
CMR: Cardiac magnetic resonance
CAG: Coronary artery angiography
ECG: Electrocardiography
TIMI: Thrombolysis in myocardial infarction
SSFP: Steady-state free precession
STIR: Short time inversion recovery
LGE: Late gadolinium enhancement
PSIR: Phase sensitive inversion recovery
LVEF: Left ventricular ejection fraction
AAR: Area at risk
SD: Standard deviations
IS: Infarct size
MSI: Myocardial salvage index
LV: Left ventricular
AUC: Area under the curve
ROC: Receiver operating characteristic
OR: Odds ratio
CI: Confidence interval
CK: Creatine kinase
CK-MB: Creatine kinase isoenzyme
PRO-BNP: Pro-B-type natriuretic peptide
CRP: C-reaction protein
LDL-C: Low density lipoprotein cholesterol
HDL-C: High density lipoprotein cholesterol
LCX: Left circumflex coronary artery
LAD: Left anterior descending artery
RCA: Right coronary artery
CAD: Coronary artery disease
